# A human rights approach to preventing racial discrimination in health care

**DOI:** 10.2471/BLT.25.293305

**Published:** 2025-08-13

**Authors:** Michal Balcerzak, Ewa Michalkiewicz-Kądziela

**Affiliations:** a Faculty of Law and Administration, University of Szczecin, ul. Narutowicza 17a, 70-240, Szczecin, Poland.

Racial discrimination, defined as any distinction, exclusion, restriction or preference based on race, colour, descent or national or ethnic origin that leads to nullifying or impairing the recognition, enjoyment or exercise, on equal footing, of human rights and freedoms,^1^ remains pervasive in health-care systems. Deeply rooted in historical injustices such as slavery and slave trade, colonialism, Jim Crow laws in the United States of America or apartheid in South Africa, racial discrimination exacerbates disparities in access to care, quality of treatment and health outcomes. Discriminatory practices based on race constitute both a moral and legal issue, and a public health threat, contributing to the prevalence of preventable diseases and even deaths.^2^^,^^3^ Evidence shows that disparities in morbidity and mortality exist across different ethnic minority groups, for instance in relation to conditions such as rheumatic heart disease, cancer, asthma, chronic pulmonary disease and diabetes.^4^ Racial discrimination contributes to current inequity and, if unaddressed, is likely to perpetuate or worsen disparities. For example, in digital health systems, unequal and biased data collection in artificial intelligence-based tools risks reproducing structural inequities among ethnic minority patient communities.^5^ In this article, we propose a practical, global and rights-based approach for addressing racial discrimination in health care, informed by international human rights standards.

Despite longstanding international efforts, racial discrimination, along with classism, sexism, ableism, xenophobia, homophobia and transphobia, remains a structural determinant that adversely influences access to health care and the right to health.^3^^,^^5^ The World Health Organization (WHO) has developed a comprehensive, evidence-based framework for understanding the social determinants of health equity, including racial discrimination.^6^ This approach integrates scientific research and policy analysis to examine the complex social, economic and environmental factors that influence health outcomes and contribute to health disparities. We argue, however, that addressing these inequities requires a principled human rights perspective to better understand the root problem of racism and seek solutions. Current approaches to racial discrimination in health systems remain unsatisfactory, as they often do not focus on preventing racial discrimination in health care, and are often fragmented and superficial, even when framed within the rhetoric of the rights of patients.

Drawing on international human rights law and public health practice, we advocate for a three-step approach to address racial discrimination in health-care systems: (i) recognizing the health-specific risks of racial discrimination; (ii) identifying enforceable human rights entitlements; and (iii) applying targeted policy interventions (Box 1). This approach is designed for national health authorities and policy-makers seeking to build accountable, equitable health-care systems. However, to move from principle to practice, a human-rights-based approach must be complemented by actionable policy interventions and measurable outcomes.

Box 1 **Suggested three-step approach to address racial discrimination in health care**

***Step one: recognizing specific health risks***

The first step is to acknowledge how structural and systemic racial discrimination manifests in health care. Racial and ethnic minority populations often face higher exposure to environmental and occupational health hazards, lack of clean water, poor housing and food insecurity.^7^ Climate-related health risks, such as heatwaves, floods and disease outbreaks, also disproportionately affect ethnic minority communities due to their geographic displacement or socioeconomic status.

Health system-level discrimination includes less access to health care compared to other groups in the society, as well as underdiagnosis, misdiagnosis or coercive treatment, especially in mental health services. Ethnic minority women and girls from Indigenous, Roma or African descent communities face compounded inequities such as coerced sterilizations, barriers to reproductive health care and cultural insensitivity.^3^ These conditions and practices reflect broader patterns of systemic exclusion and must be recognized as specific human rights risks.

***Step two: identifying human rights entitlements***

This step links the health risks of racial discrimination to State obligations under international human rights law. The right to health is articulated in multiple treaties, including the 1965 International Convention on the Elimination of All Forms of Racial Discrimination, the 1966 International Covenant on Social and Cultural Rights or the 1989 Convention on the Rights of the Child. From these and other international standards, such as those expressed in the Committee on the Elimination of Racial Discrimination’s General Recommendation No. 37 (2024),^3^^,^^8^ several enforceable rights emerge, in particular:

- Protection from avoidable harm: States must ensure safe environments and mitigate risk factors disproportionately affecting marginalized ethnic minority groups.
- Bodily autonomy and informed consent: all individuals have the right to participate in health-care decisions, especially in reproductive and mental health contexts.
- Access to information: this includes culturally competent, linguistically accessible and accurate health literacy resources.
- Participation: affected communities should meaningfully participate in shaping health policies at the national and international levels.
- Effective remedies: victims of racial discrimination must have access to restitution and compensation, if their rights have been violated.

***Step three: applying targeted and actionable policies***

The third step implements rights of particular importance for preventing and eradicating racial discrimination in health care through national policies and tangible actions. Drawing from the Committee on the Elimination of Racial Discrimination’s General Recommendation No. 37 (2024)^3^ and the 2022 report of the United Nations Special Rapporteur on health,^7^ we suggest that actionable policies should specifically include:

- Legislation: enact laws prohibiting racial discrimination and mandating equity impact assessments in health care.
- Planning: develop and fund national health equity strategies.
- Hiring and workforce development: introduce equity-oriented hiring practices across the health-care sector.
- Training: embed anti-racism and equity curricula in medical education.
- Technology oversight: ensure that artificial intelligence and digital tools are audited for racial bias.
- Data safeguards: use demographic data ethically to measure disparities, not reinforce them.

## Incentives for improvement

While international standards should serve as a normative backbone of anti-racism strategies in health care, pragmatic incentives are essential for motivating sustained action and policy innovation. From a public health standpoint, addressing ethnic and racial disparities improves overall population health. When marginalized groups receive appropriate, culturally responsive care, chronic disease burdens are reduced, emergency care reliance decreases and preventive health behaviours increase.^9^ This enhances individual well-being and alleviates systemic strain on national health systems, freeing up resources and improving efficiency. Economically, equitable health-care systems are more productive and resilient.^10^ Social cohesion is another critical incentive. Addressing racial inequities fosters trust in public institutions and reduces the risk of social unrest, particularly in multiethnic societies. When health-care systems are seen as fair, inclusive and racism-free, they gain legitimacy and strengthen the social contract between the State and its citizens. Furthermore, reputation also matters. Countries that comply with international human rights norms and actively promote ethnic and racial equity are viewed as more trustworthy and responsible on the global stage.

## Implementation in practice

Despite persistent shortcomings in many governments’ efforts to prevent racial discrimination and promote equity in health care,^11^ some progress has been made, particularly in areas such as hiring and workforce. Countries such as Canada (2023 Employment Equity Act Review Report), New Zealand (2020 Public Service Act) and the United Kingdom of Great Britain and Northern Ireland (2023 National Health Service Equality, Diversity and Inclusion Improvement Plan) have elaborated strategies to diversify health-care hiring, set equity targets and monitor recruitment data. In the United States, institutions that have adopted racial equity programmes report improved diversity in staffing, increased patient satisfaction and reduced disparities in chronic disease outcomes.^12^

Beyond workforce representation, several countries have taken practical steps to integrate anti-racism into health-care delivery, service design and patient engagement. For example, in South Africa, the health department developed community health worker models targeting underserved racial groups, resulting in higher immunization rates, improved maternal health services and more culturally aligned health-care delivery in rural and township areas.^13^ Similarly, Brazil’s Unified Health System has recognized and integrated Afro-Brazilian traditional medicine into primary care services, enhancing accessibility and legitimacy for Afro-descendant populations (National Policy on Integrative and Complementary Practices). In Australia, the government has supported the roll-out of Aboriginal Community Controlled Health Services, which are governed and operated by Indigenous communities. These services address social determinants of health and improve trust and continuity of care, contributing to measurable reductions in preventable hospitalizations and child mortality among Aboriginal populations.^14^

These developments illustrate how both targeted hiring and broader structural changes in care delivery, governance and education can operationalize anti-racism principles in health care. Although challenges remain, these practices offer replicable models for institutional change and help implement the international human rights obligations in advancing racial equity in health systems.

## Conclusions

United Nations bodies such as the Committee on the Elimination of Racial Discrimination,^3^ the Special Rapporteur on health^11^ and WHO^6^ provide essential guidance on preventing and addressing racial discrimination in health care based on countries’ international obligations, but implementation lies with national governments and local institutions. Adherence to international human rights norms, assisted by the three-step approach presented in this article would enable the development of racism-conscious, accountable, inclusive and equitable health-care systems. To advance this agenda, stakeholders must adopt enforceable measures that dismantle structural racism in health care. Integrating rights-based policies with operational frameworks and meaningful participation of minority ethnic and racial groups offers a pragmatic path towards health equity. The success of these efforts will be measured not only in laws passed, but in lives improved and disparities narrowed.
